# The bone morphogenetic protein antagonist gremlin 1 is overexpressed in human cancers and interacts with YWHAH protein

**DOI:** 10.1186/1471-2407-6-74

**Published:** 2006-03-18

**Authors:** Hong Namkoong, Seung Min Shin, Hyun Kee Kim, Seon-Ah Ha, Goang Won Cho, Soo Young Hur, Tae Eung Kim, Jin Woo Kim

**Affiliations:** 1 Molecular Genetic Laboratory, College of Medicine, The Catholic University of Korea, Seoul 137-040, Korea; 2 Department of Obstetrics and Gynecology, College of Medicine, The Catholic University of Korea, Seoul 137-040, Korea

## Abstract

**Background:**

Basic studies of oncogenesis have demonstrated that either the elevated production of particular oncogene proteins or the occurrence of qualitative abnormalities in oncogenes can contribute to neoplastic cellular transformation. The purpose of our study was to identify an unique gene that shows cancer-associated expression, and characterizes its function related to human carcinogenesis.

**Methods:**

We used the differential display (DD) RT-PCR method using normal cervical, cervical cancer, metastatic cervical tissues, and cervical cancer cell lines to identify genes overexpressed in cervical cancers and identified gremlin 1 which was overexpressed in cervical cancers. We determined expression levels of gremlin 1 using Northern blot analysis and immunohistochemical study in various types of human normal and cancer tissues. To understand the tumorigenesis pathway of identified gremlin 1 protein, we performed a yeast two-hybrid screen, GST pull down assay, and immunoprecipitation to identify gremlin 1 interacting proteins.

**Results:**

DDRT-PCR analysis revealed that gremlin 1 was overexpressed in uterine cervical cancer. We also identified a human gremlin 1 that was overexpressed in various human tumors including carcinomas of the lung, ovary, kidney, breast, colon, pancreas, and sarcoma. PIG-2-transfected HEK 293 cells exhibited growth stimulation and increased telomerase activity. Gremlin 1 interacted with homo sapiens tyrosine 3-monooxygenase/tryptophan 5-monooxygenase activation protein, eta polypeptide (14-3-3 eta; YWHAH). YWHAH protein binding site for gremlin 1 was located between residues 61–80 and gremlin 1 binding site for YWHAH was found to be located between residues 1 to 67.

**Conclusion:**

Gremlin 1 may play an oncogenic role especially in carcinomas of the uterine cervix, lung, ovary, kidney, breast, colon, pancreas, and sarcoma. Over-expressed gremlin 1 functions by interaction with YWHAH. Therefore, Gremlin 1 and its binding protein YWHAH could be good targets for developing diagnostic and therapeutic strategies against human cancers.

## Background

The identification of molecular alterations in cancerous and pre-cancerous cells has provided insight into the role of oncogenes and tumor suppressor genes in tumor initiation and progression [1]. Oncogenes are derived from highly conserved proto-oncogenes that are altered by chromosomal point mutations, gene amplifications, or gene rearrangements [2]. Structural alteration of proto-oncogenes leads to a quantitative or qualitative change in the expression of the corresponding protein product. The signal transduction pathways subverted by oncoproteins govern fundamental cell functions, including proliferation, cell cycle regulation, and apoptosis [3].

Although genetic characterization of tumor tissues demonstrates that mutation of the *p53 *gene is the most common genetic alteration in human cancers, the mutation ratio of the *p53 *gene in uterine cervical cancer is relatively low [4,5]. It suggests that there are other oncogenes involved in cervical carcinogenesis. We applied the DDRT-PCR method to discover genes involved in tumorigenesis of human cervical tissue, and identified the new human cervical cancer-related gene, proliferation-inducing gene 2 (*PIG-2*) (GenBank accession number AY232290), which exhibits close similarity to gremlin 1 cDNA (GenBank accession number NM_013372) in the database.

The Drm (also known as gremlin) and its independently isolated Xenopus homolog, gremlin, a 184-aa protein initially identified through differential screening as a transcriptional down-regulated gene in v-*mos*-transformed rat embryonic fibroblasts [6], belongs to the Dan family of secreted glycosylated proteins [7,8], which contains a highly conserved cysteine knot domain shared by the TGF-β superfamily, PDGF, nerve growth factor, and other secreted proteins [9]. Drm and Dan regulate early development [10-13], tumorigenesis [6,14,15], and renal pathophysiology [16].

Gremlin gene encodes a member of the bone morphogenic protein (BMP) antagonist family. Like BMPs, BMP antagonists contain cystine knots and typically form homo- and heterodimers. The cerberus and dan subfamily of BMP antagonists, to which this gene belongs, is characterized by a C-terminal cystine knot with an eight-membered ring. The antagonistic effect of the secreted glycosylated protein encoded by this gene is likely due to its direct binding to BMP proteins. As an antagonist of BMP, this gene may play a role in regulating organogenesis, body patterning, and tissue differentiation. In mouse, this protein has been shown to relay the sonic hedgehog signal from the polarizing region to the apical ectodermal ridge during limb bud outgrowth [17].

The action of Drm and Dan on development and possibly diabetic nephropathy is mediated by heterodimerizing with certain BMPs [9], in particular BMP2, 4, and 7 [7,8,16,18] to subsequently block the ability of BMPs to bind their receptors [7,18,19]. Chen et al. have previously shown that the capacity of Drm to suppress transformation and tumorigenesis [6,14,15] is mediated by a mechanism that is independent of BMPs and involves both up-regulation of p21^*Cip*1 ^and down-regulation of p42/44 MAPK [14], suggesting additional target(s) for Drm and other Dan family members.

To identify additional target proteins for PIG-2 which exhibits close similarity to gremlin 1, we used a yeast two-hybrid screening approach with a PIG-2-LexA fusion construct as the bait to search for potential PIG2-binding partners. This approach identified homo sapiens tyrosine 3-monooxygenase/tryptophan 5-monooxygenase activation protein, eta polypeptide (14-3-3 eta; YWHAH) [20-28] as one type of the PIG-2-interacting proteins. Thus, the data demonstrate that Gremlin 1 functionally interacts with YWHAH protein to act as an oncoprotein for the genesis of human cancers.

## Methods

### Tissues and cell lines

For Differential display (DD) of mRNA, normal exocervical tissue specimen was obtained from uterine myoma patients during hysterectomy and untreated primary cervical cancer tissues and metastatic lymph node tissues were obtained during radical hysterectomy. Patient consent was obtained from each individual and the use of tissue samples was approved by the ethics committee of our institution. The cervical caner cell line used in DD was CasKi and CUMC-6 which was isolated in our laboratory and maintained as previously described [29]. Mammalian cell lines described below were all obtained from the American Type Culture Collection (ATCC; Manassas, VA, USA): CasKi is a human cervical cancer cell line. NCI-H441, NCI-H157, and NCI-H2009 are human lung cancer cell lines.

### DDRT-PCR

Total RNA was extracted from tissues and cells using RNA extraction kit (RNeasy total RNA kit; Qiagen Inc., Valencia, CA) and 0.2 μg of total RNA was used to generate cDNA in reverse transcription reaction (RNAimage™ kit, GenHunter, MA). With the use of the differential display kit (RNAimage™ kit), we performed PCR using oligo-dT primers and arbitrary sequences, each 13 bases in length according to the manufacturer's recommendations [30]. After cDNAs of 3' termini of mRNAs were generated, the PCR products were separated by electrophoreses on a 6% denaturing polyacrylamide gel. Bands representing cDNAs of interest were excised from dried sequencing gel. The cDNAs were eluted in distilled water by boiling for 15 minutes and then were reamplified without [α-^35^S]dATP, and with 20 μM dNTPs instead of 2 μM dNTPs. From the films, a 282 bp cDNA (referred to as CC282) was identified that was expressed in primary cervical cancer tissue, metastatic lymph node and cervical cancer cell lines but not in normal cervical tissue. CC282 was identified by the use of 5' arbitrary primer H-AP28 (5' -AAGCTTACGATGC-3') and 3' H-T_11_C anchored primer (5' -AAGCTTTTTTTTTTTC-3') (GenHunter). CC282 was then subcloned into pGEM-T easy vector with the use of the TA cloning system and sequenced with the use of the Sequenase Version 2.0 DNA Sequencing System (United States Biochemical Co., Cleveland, OH).

### Northern blot analysis

Total RNA was extracted from fresh human tissues and cell lines using RNeasy total RNA kit (Qiagen). Northern blot analysis was carried out, in which 20 μg of denatured total RNA was electrophoresed on a 1.0% formaldehyde agarose gel and transferred to nylon membrane (Roche Diagnostics GmbH, Mannheim, Germany). The mRNA expression of *PIG-2 *was also assessed in normal human tissues and a variety of human cancer cell lines with the use of prepared membranes obtained from Clontech (Palo Alto, CA) and processed as recommended by the supplier. Human β-actin cDNA control probe provided by Clontech was used as a loading control. All blots were hybridized with the randomly primed [^32^P]-labeled *PIG-2 *partial cDNA probe (the CC282 fragment).

### Growth curve

To test the effect of PIG-2 gene on HEK 293 cell growth, 1 × 10^5 ^wild-type HEK 293 cells, PIG-2 gene transfected HEK 293 cells, and HEK 293 cells transfected with pcDNA3.1 alone were cultured for 13 days. In three independent experiments, cells in triplicate flasks were detached and viable cells counted every other day using trypan blue dye exclusion.

### Transformation and morphology

*PIG-2*-transfected HEK 293 cells were maintained in culture for 4–5 weeks with the corresponding media replaced every 3 days and monitored microscopically. HEK 293 nontransformed cells were seeded in parallel. To examine cell morphologies, clones of HEK 293 cells stably transfected with the *PIG-2 *gene were grown to approximately 70% of confluency in culture flasks, and photographed by Olympus (Inha, Japan) phase-contrast microscopy (magnification, **× **100).

### Immunohistochemistry

For immunohistochemistry, cryosections (5 μm thick) of human normal and cancer tissues were used. The sections were deparaffinized with xylene and ethanol. After washing with tap water, the sections were treated with methanolic H_2_O_2 _for 30 minutes. Before incubation with primary antibody, the sections were permeabilized by incubation in 0.5% Triton X-100 in phosphate-buffered saline (PBS) for 15 minutes and then blocked with normal goat serum for 15 minutes. The sections were incubated with polyclonal anti-Gremlin antibody (IMGENEX, San Diego, CA) for 2 hours at room temperature. After three washes with PBS, the sections were sequentially incubated with biotinylated species-specific secondary antibodies (Vector Laboratories, Burlington, CA) for 1 hour at room temperature, and then avidin and biotinylated horseradish peroxidase according to the manufacturer's recommendations. Aminoethyl carbozole (AEC) was used as the chromogen. After immunostaining, sections were counterstained with hematoxylin. Sections were photographed on an Olympus photomicroscope (Inha, Japan).

### Telomerase activity assay

Telomerase activity was measured with the Telo *TAGGG *Telomerase PCR-ELISA kit (Roche, Germany). The kit provides an immortalized human 293 kidney cell extract as a positive control and 293 cell extract pretreated with RNase as a negative control. All the experiments were performed in triplicate.

### Yeast two-hybrid screening and β-galactosidase assay

The MATCHMAKER LexA two-hybrid system was used to identify proteins from the human fetal brain MATCHMAKER cDNA library that could bind a PIG-2 fusion protein (Clontech, Palo Alto, CA). All experiments were performed in the yeast strain EGY48 transformed with p8op-lacZ, which expresses lacZ and leu genes as reporters (Clontech). We inserted a PIG-2 cDNA fragment into a yeast two-hybrid vector (pLexA) (Clontech) containing the LexA DNA-binding domain. Yeast cells expressing the LexA-PIG-2 were transformed with a human fetal brain cDNA library (Invitrogen) that expresses B42AD fusion proteins. After library transformation, cells are plated on minimal synthetic dropout non-induction medium (Sigma) that selects for both the bait (PIG-2) and the AD/library plasmids to improve the chances of detecting AD fusion proteins. To confirm the interaction between PIG-2 and binding protein YWHAH, plasmids expressing PIG-2 and YWHAH were co-transformed into yeast cells. β-galactosidase filter lift assays were performed by replica-plating the co-transformants expressing PIG-2 and YWHAH on Trp-, Leu-, His- selection plates. We used a yeast mating assay to eliminate false positive interactions.

### GST-tagged YWHAH protein expression and pull down experiments

GST-tagged proteins were expressed and extracts were prepared as recommended by the manufacturer (Amersham Pharmacia Biotech). *E.coli *(strain BL21) extracts containing GST alone, GST-YWHAH and PIG-2 deletion mutants or GST-PIG-2 and YWHAH deletion mutants were incubated with 30 μl of glutathione-sepharose in 500 μl of lysis buffer (20 mM Tris-HCl [pH 6.8], 50 mM NaCl, 0.1 mM EDTA, 0.1% Triton X-1000, containing 1 mM phenylmethylsulfonyl fluoride, 5 mM dithiothreitol, 1 mg/ml leupeptin, 1 mg/ml aprotinin, and 1.5 mg/ml pepstatin) for 12 hours at 4°C. Fusion proteins bound to sepharose beads were quantitated by Commassie blue staining on SDS-polyacrylamide gels. The fusion protein-bound beads were washed three times with 500 μl of TEN buffer (20 mM Tris HCl [pH 7.4], 0.1 mM EDTA, 100 mM NaCl). For GST pull down experiments, fusion proteins bound to the beads were incubated with proteins from HEK 293 total cell extracts expressing PIG-2 or YWHAH for 12 hours at 4?. The beads were washed using NETN buffer (0.5% Nonidet P-40, 0.1 mM EDTA, 20 mM Tris HCl [pH 7.4], 300 mM NaCl) and eluted with 30 ml of SDS sample buffer (75 mM Tris-HCl [pH 6.8], 0.5% glycerol, 1% SDS, 4% mercaptoethanol, 0.01% bromophenol blue), and boiled for 3 minutes before separating on an 10% SDS-polyacrylamide gel. Eluted proteins were subjected to western blot analysis.

### Immunoprecipitation and Western blot

To confirm the interaction between PIG-2 and binding protein YWHAH, HEK 293 cells were co-transfected with pFLAG-CMV (Invitrogen, Carlsbad, CA) encoding the full-length of PIG-2 and pcDNA3.1-Myc-His/YWHAH (Invitrogen). After 48 hours, the cells were harvested and lysed with RIPA buffer (20 mM Hepes [pH 7.2], 1% Triton X-100, 1% sodium deoxycholate, 0.2% SDS, 150 mM NaCl, 1 mM Na3VO4, 1 mM NaF, 10% glycerol, 10 g/ml leupeptin, 10 g/ml aprotinin, 1 mM phenymethylsulfonyl fluoride). The lysates were precleared with preimmune serum (mouse) and protein A-Sepharose at 4°C for 30 minutes. Protein concentrations were determined using the BioRad Protein Assay (Bio-Rad, Hercules, CA) with bovine serum albumin as a standard. Aliquots (1 mg) of precleared cell lysates were incubated with a 1:500 dilution of anti-FLAG (Sigma, F3165) or 1:500 dilution of anti-Myc (Santa Cruz, 9E10) monoclonal antibody (mAb) and 40 ml of a 1:1 slurry of protein A-Sepharose beads in PBS for 4 hours at 4°C. The immune complexes were collected by centrifugation (2,000 × g for 5 minutes at 4°C), washed five times with a buffer (20 mM Tris, pH 7.5, 1 mM EDTA, 1 mM EGTA, 150 mM NaCl, 2 mM Na3VO4, 10% glycerol and 1 % Nonidet P-40), and subjected to SDS-PAGE (10%~12%). Separated proteins were transferred to nitrocellulose membranes (Schleicher and schuell), blocked with 5% nonfat dry milk in TBST buffer (20 mM tris-HCl [pH 7.6], 150 mM NaCl, and 0.5% Tween20) for 2 hours at room temperature. Blot washed two times in TBST buffer, primary antibodies incubated with a 1:1000 dilution of anti-Myc or 1:4000 dilution of anti-FLAG antibodies in TBST for overnight at 4°C. Blot was washed three times in TBST, and then incubated with goat anti-mouse secondary antibody conjugated to horseradish peroxidase (Sigma), and washed four times in TBST. Bound protein visualized using the Super signal west pico chemiluminescent substrate (PIERCE) followed by autoradiography for 15 seconds~60 minutes.

## Results

### Identification of the PIG-2 gene and different expressions of PIG-2 in human cervical tissues

**Figure 1:**
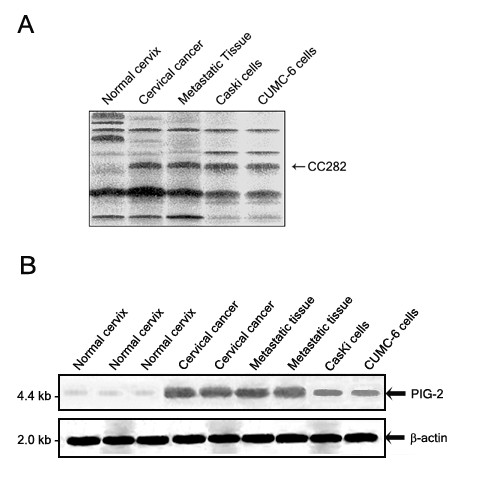
**Identification and expression level of *PIG-2*. A**) Comparison gene expression profiles by DDRT-PCR from total RNA isolated from normal cervical tissue, primary cervical cancer, cervical cancer tissue metastatic to lymph node and from CasKi and CUMC-6 cervical cancer cell lines. Differential display was carried out 5' arbitrary primer H-AP28 (5' -AAGCTTACGATGC-3') and 3' H-T_11_C (5' -AAGCTTTTTTTTTTTC-3'). The PCR products were resolved by electrophoresis. CC282 is the name of the partial *PIG-2 *gene product. The arrow identifies the location relative to other PCR products. (**B**) Total RNAs were isolated from normal cervical tissue, primary cervical cancer, cervical cancer tissue metastatic to lymph node and from CasKi andCUMC-6 cervical cancer cell lines. Blot was hybridized with the randomly primed [^32^P]-labeled *PIG-2 *partial cDNA probe (the CC282 fragment). Human β-actin cDNA was used as a control probe (lower panel).

To identify oncogenes that may be responsible for the formation of cervical and other cancers, we used differential display RT-PCR and identified the 200 bp partial cDNA fragment CC282. As shown in Figure 1A, among the multiple bands, CC282 was strongly expressed in cervical cancer tissue, metastatic lymph node tissue, and CasKi and CUMC-6 cervical cancer cells, but not normal cervical tissue. Using the partial cDNA CC282 as a probe, we screened normal human lung, normal uterine cervical tissue, and cervical cancer cDNA library to identify the full-length cDNA. One clone with size of 4151 bp was isolated from the human lung cDNA library. This clone, named *PIG-2 *for human proliferation-inducing gene 2 (GenBank accession number AY 232290), encoded a polypeptide of 184 amino acids with a predicted relative molecular mass of 21 kDa. *PIG-2 *exhibited 99% homology with the recorded human gremlin 1, cysteine knot superfamily, homolog (Xenopus laevis) (GREM1), mRNA sequence (GenBank accession number NM_013372). The CC282 partial cDNA identified in the differential display corresponded to nucleotides 3901–4100 of *PIG-2*. Next, we performed Northern blot analysis to examine the expression of *PIG-2 *in normal cervical tissues, cervical cancer tissues, metastatic lymph node tissues, and cervical cancer cell lines human tissue using the CC282 partial cDNA fragment. *PIG-2 *(~4.5 kb) mRNA was strongly expressed in cervical cancer tissue, metastatic lymph node tissue, and cervical cancer cells. I normal cervical tissues, the expression of *PIG-2 *was very low (Figure 1B).

### Differential expression of PIG-2 in various types of human normal and cancer tissues

**Figure 2:**
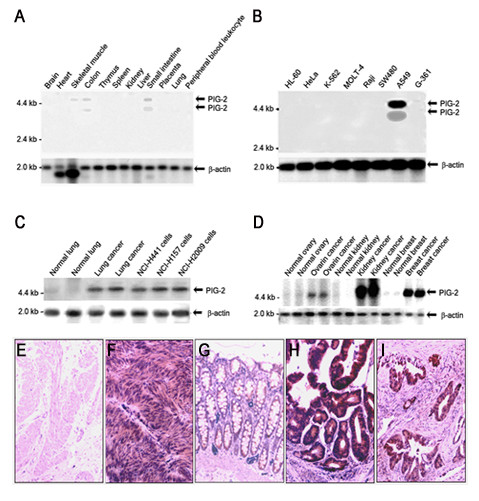
**Expression of *PIG-2 *in human tissues by Northern analyses and immunohistochemical studies. **Northern blotting analyses were performed to determine the expression of *PIG-2 *in different human tissues. Normal 12 lane multiple tissue northern blot (**A**) or human cancer cell line multiple northern-blot purchased from Clontech (**B**) was probed with a radioactively labeled CC282 partial cDNA (upper panel) or human β-actin cDNA control probe provided by Clontech (lower panel). (**C**) Total RNAs were isolated from normal lung tissue, primary lung cancer and from NCI-H441, NCI-H157 and NCI-H2009 lung cancer cell lines. Blot was hybridized with the randomly primed [^32^P]-labeled *PIG-2 *partial cDNA probe (the CC282 fragment). Human β-actin cDNA was used as a control probe (lower panel). (**D**) Comparison of *PIG-2 *mRNA expression in human tumor tissues and their corresponding normal counterparts (upper panel). Total RNAs were extracted from fresh human normal and cancer tissues. The same blot was probed with β-actin as a loading control (lower panel). (**E-I**) Immunohistochemical staining for PIG-2 expression of human normal muscle (**E**), leiomyosarcoma (**F**), normal colon tissue (**G**), colon cancer (**H**), and pancrease cancer tissue (**I**), Original magnification, × 100.

Northern blot analysis was performed to examine the expression of *PIG-2 *in human tissue using the CC282 partial cDNA fragment. A transcript of approximately 4.5 kb is present in skeletal muscle, colon, and small intestine tissues examined, as well as a weaker band at 3.7 kb in colon and small intestine (Figure 2A). The levels of mRNA expression were quantified by comparison with the levels of expression of β-actin. We also examined the expression of *PIG-2 *in several cancer cell lines. *PIG-2 *mRNA was overexpressed only in human lung cancer cell line A549. A dominant transcript of 4.5 kb is present in lung cancer cells. In addition, a transcript of approximately 3.7 kb was also present in A549 lung cancer cells (Figure 2B). There was no expression of *PIG-2 *in promyelocytic leukemia cell line HL-60, human cervical cancer cell line HeLa, chronic myelogenous leukemia cell line K-562, lymphoblastic leukemia cell line MOLT-4, Burkitt's lymphoma cell line Raji, colon cancer cell line SW480, and melanoma line G361. Because *PIG-2 *was overexpressed only in A549 lung cancer cells among 8 cancer cell lines tested, we studied the expression levels of *PIG-2 *in normal lung tissues, fresh primary lung cancer tissues, and lung cancer cell lines (NCI-H441, NCI-H157 and NCI-H2009). Northern blot analysis revealed that fresh primary human lung cancer tissues and lung cancer cell lines showed increased expression of *PIG-2 *levels compared to normal lung tissues (Figure 2C). Northern blot analysis also revealed that fresh primary human tumor tissues, including carcinomas of the ovary, kidney and breast, showed increased expression of *PIG-2 *when compared with their normal counterparts (Figure 2D).

Using anti-gremlin polyclonal antibody, normal or cancer tissue from muscle, colon and pancreas were subjected to immunohistochemical experiments. As a result, PIG-2 was over-expressed in all muscle, colon and pancreas cancer tissues. The immunoreactivity was observed mainly in cancer cells with a cytoplasm dominant manner (Figures 2F, 2H and 2I). In corresponding normal muscle and colon tissues, there were very weak expressions of PIG-2 (Figures 2E and 2G). Although further investigation with larger number of samples will be needed, these results indicate that increased expression of the PIG-2 may be associated with human carcinogenesis.

### Morphological changes of HEK 293 cells after transfection with PIG-2 gene

**Figure 3:**
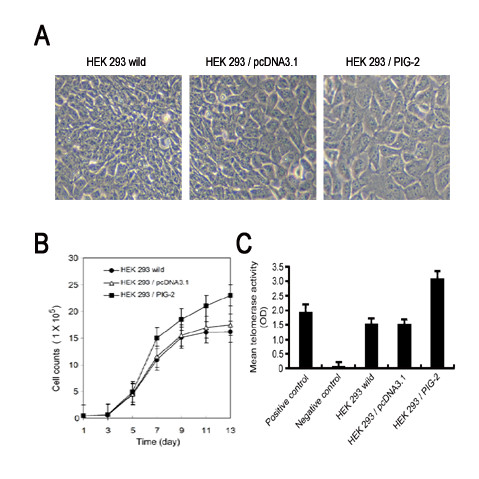
**Morphology, growth curve and telomerase activity in *PIG-2*-transfected HEK 293 cells. **(**A**) Phase-contrast features of wild-type HEK 293 cells (left panel), cells transfected vector alone (middle panel), and cells transfected with PIG-2 gene (left panel). Magnifications, **× **100.(**B**) Growth stimulatory effect of PIG-2 in HEK 293 cells. PIG-2 induces growth stimulation in 293 cells. Viability of 293 cells after transfection with PIG-2 or pcDNA3.1 vector alone, respectively. The data are the number of viable cells grown for 13 days in culture and represent the mean ± S.D. of triplicate determinations. (**C**) Determination of telomerase activity. Human telomerase-positive embryonic kidney 293 cells, 293 cell extracts treated with RNase (+ RNase), wild-type 293 cells, cells transfected with vector alone and cells transfected with PIG-2 were analyzed by Telomerase PCR ELISA. Assays were performed according to the kit protocol with amounts of extracts equivalent to 1 × 10^3 ^cells. The telomerase activity in 293 cells, which served as a positive control, was abolished by pretreatment with RNase. Results are the average mean absorbance values from three separate experiments (means and 95% confidence intervals).

Human embryonic kidney (HEK) 293 cells, a differentiated fibroblast cell line, is a spindle shaped fibroblast cell having a long slender nucleus and a scanty amount of cytoplasm (Figure 3A; left panel). There were no discernable differences in cell morphology between wild-type HEK 293 cells and cells transfected with vector alone (Figure 3A; middle panel). But HEK 293 cells expressing PIG-2 are altered to polygonal cells with an ovoid nucleus and plump cytoplasm (Figure 3A; right panel).

### Growth stimulation of HEK 293 cells by PIG-2

The rate of PIG-2-transfected HEK 293 cell growth was increased compared to those of cells transfected with vector alone or wild-type HEK 293 cells. About 150% of PIG-2-transfected HEK 293 cells remained viable at 13 days when compared with wild-type HEK 293 cells (Figure 3B).

### Telomerase activity in the PIG-2-transfected HEK 293 cells

Alterations in telomere biology both suppress and facilitate malignant transformation by regulating genomic stability and cellular life span [31]. Telomerase is an enzymatic ribonucleoprotein complex that acts as a reverse transcriptase in the elongation of telomeres. Telomerase activity is almost absent in somatic cells, but it is detected in embryonic stem cells and in the vast majority of tumor cells [32]. To explain the possible oncogenic role of *PIG-2*-transfected cells, we determined telomerase activity in PIG-2-transfected HEK 293 cells. Wild-type HEK 293 cells showed detectable telomerase activity (Figure 3C). *PIG-2 *gene transfection increased telomerase activity up to about 2-fold when compared with HEK 293 wild-type cells (Figure 3C).

### PIG-2 interacts with YWHAH in vivo

**Figure 4:**
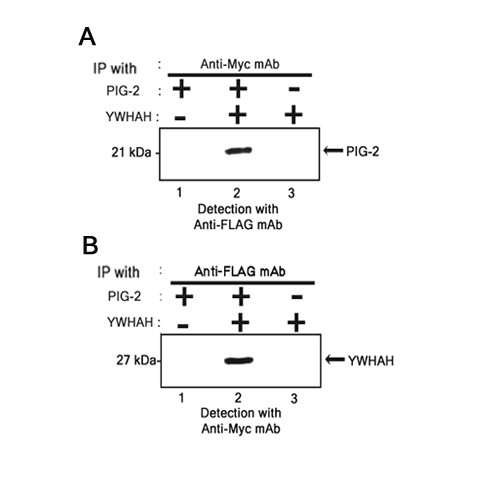
**Protein-interaction of PIG-2. **(**A**)PIG-2 binds to YWHAH. Co-immunoprecipitation from transfected HEK 293 cells producing PIG-2 and YWHAH proteins was done. Immunoprecipitation was performed with anti-Myc mAb. The protein in the pellets was detected with anti-FLAG mAb. (**B**) YWHAH binds to PIG-2. Co-immunoprecipitation from transfected HEK 293 cell lines producing YWHAH and PIG-2 proteins was done. Immunoprecipitation was performed with anti-FLAG mAb. The protein in the pellets was detected with anti-Myc mAb.

To understand the PIG-2 tumorigenesis pathway, we performed a yeast two-hybrid screen and identified the YWHAH protein, encoded by the gene *homo sapiens tyrosine 3-monooxygenase/tryptophan 5-monooxygenase activation protein, eta polypeptide *(GenBank accession number NM_003405), was interacted with PIG-2. The interaction of PIG-2 and YWHAH identified by yeast two hybrid screen was confirmed by glutathione-S-transferase (GST) pull-down experiments and immunoprecipitation studies *in vitro*. The physical interaction between PIG-2 and YWHAH was examined *in vivo *by co-immunoprecipitation in HEK 293 cells. The FLAG epitope-tagged PIG-2 was transiently overexpressed together with the Myc-tagged YWHAH in HEK 293 cells, and the cell lysates were immunoprecipitated with the anti-Myc antibody and detected by western blotting with anti-FLAG antibody (Figure 4A). We also performed the immunoprecipitation of reverse form of co-expression, YWHAH-Myc and PIG-2-FLAG proteins, *in vivo *(Figure 4B). As shown in Figure 4A, PIG-2 could be co-immunoprecipitated with YWHAH, indicating that YWHAH is likely to be a binding partner. Taken together, co-immunoprecipitation showed that PIG-2 binds YWHAH protein (Figure 4).

### GST full down assays

**Figure 5:**
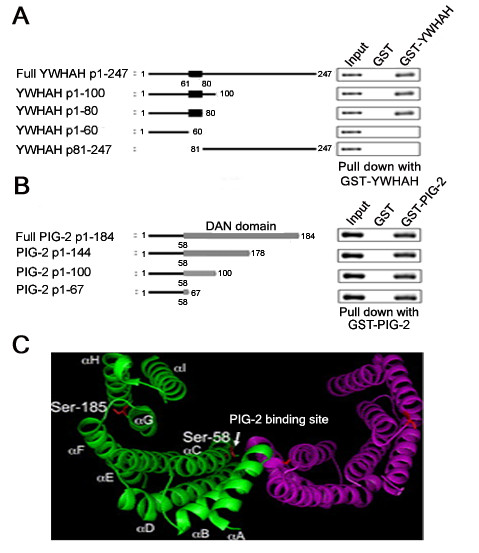
**Association of PIG-2 and YWHAH**. (**A**) Mapping of PIG-2 binding domain of YWHAH. Five GST fusion constructs, Full-YWHAHp1-247, YWHAHp1-100, YWHAHp1–80, YWHAHp1–60 and YWHAHp81–247 were prepared using YWHAH cDNA as a template DNA. The GST pull-down assay showed that Three GST-YWHAH constructs, Full-YWHAHp1–247, YWHAHp1–100 and YWHAHp1–80 bind YWHAH, but not YWHAHp1–60 and YWHAHp60–247. The YWHAH binding site for PIG-2 was delineated to be residues 61–80 (black box). (**B**) Mapping of YWHAH binding domain of PIG-2. Four GST fusion constructs, Full-PIG-2p1-184, PIG-2p1-144, PIG-2p1-100 and PIG-2p1-67 were prepared using PIG-2 cDNA as a template DNA. The GST pull-down assay showed all constructs, The PIG-2 binding site for YWHAH was delineated to be residues 1–67. Gray box is DAN domain. (**C**) Schematic diagram shows X-ray crystallography of YWHAH protein. PIG-2 protein binding site was indicated by arrow.

To investigate the domain of interaction between PIG-2 and YWHAH, we have used four GST fusion constructs, YWHAH (1–60), YWHAH (1–80), YWHAH (1–100), and YWHAH (81–247), prepared using YWHAH cDNA as template DNA, and by PCR amplification. Consistent with the yeast-two hybrid results, the GST pull-down assay (Figure 5A) showed that two GST-YWHAH constructs, YWHAH (1–80) and YWHAH (1–100) bind PIG-2, but not GST alone, YWHAH (1–60) and YWHAH (81–247). To further confirm the direct interaction of PIG-2 and YWHAH *in vitro*, the GST pull-down assays were performed with the GST-tagged PIG-2 proteins and the lysates of HEK 293 cells expressing full-length YWHAH were incubated with the resin-bound GST-PIG-2 proteins, and the pulled down samples were detected using anti-Myc antibodies. The GST pull-down assay (Figure 5B) showed that GST-PIG-2 (1–67), GST-PIG-2 (1–100) and GST-PIG-2 (1–147), but not GST alone, selectively pulled down with the full-length YWHAH. The PIG-2 binding site for YWHAH was found to be located between residues 1 to 67, which is not included in the DAN domain, may play an important role in the YWHAH-specific interaction as a novel protein binding domain. Therefore, the binding analysis indicates that the YWHAH binding site for PIG-2 is located between residues 61–80, which constructively include in the α helix C-α helix D linker, may play an important role in this intracellular interaction (Figure 5C).

## Discussion

To discover genes involved in human cervical carcinogenesis, we applied DDRT-PCR and identified the candidate human cervical cancer-related gene, proliferation-inducing gene 2 (*PIG-2*) (GenBank accession number AY232290). PIG-2 exhibited close similarity (99%) to gremlin 1 cDNA (GenBank accession number NM_013372) in the database.

Drm/Gremlin and Dan, two homologous secreted antagonists of bone morphogenic proteins, have been shown to regulate early development, tumorigenesis, and renal pathophysiology [6,10-16]. Topol et al. had previously shown that most tumor-derived cells fail to express Drm [33] and that in fibroblasts Drm expression is inhibited following oncogene-induced transformation [6]. Human Drm maps to chromosome 15q13-q15, within a region whose loss is associated with metastatic breast cancer and other metastatic carcinomas [34]. These properties suggested that Drm might play an inhibitory role in cell transformation or tumorigenesis. They also demonstrated that overexpression of Drm in the tumor-derived cell lines Daoy (primitive neuroectodermal) and Saos-2 (osteoblastic) significantly inhibited tumorigenesis and provided evidence that Drm can function as a novel transformation suppressor and suggested that this may occur through its affect on the levels of p21^*Cip*1 ^and phosphorylated p42/44 MAPK [14,35]. Recent publication also demonstrated that gremlin mRNA is expressed in non-malignant epithelial cells and lost in many human cancer cell lines via promoter methylation [36]. Similar finding is also reported by other group [31].

On the contrary, our experiments showed that *PIG-2 *which is identical with gremlin 1 was overexpressed in various human tumors including carcinomas of the cervix, lung, ovary, kidney, breast, colon, pancreas and sarcoma. However, expression of PIG-2 was generally down-regulated in diverse human normal tisues. In our experiments, PIG-2-transfected HEK 293 cells exhibited growth stimulation and increased telomerase activity. Although further investigation with larger number of samples will be needed, it suggests PIG-2 may play a fundamental oncogenic role in multiple body organs.

However, it is unknown how *PIG-2 *contributes to the cellular and biochemical mechanisms of human tumorigenesis. In this study, we identified an oncogene that is expressed in multiple different human cancers, and investigated whether the oncogene is responsible for the genesis of human cancer. To understand the PIG-2 tumorigenesis pathway, we performed a yeast two-hybrid screen and identified the 14-3-3 eta (YWHAH) protein was interacted with PIG-2 [20-28].

This gene product belongs to the 14-3-3 family of proteins which mediate signal transduction by binding to phosphoserine-containing proteins. This highly conserved protein family is found in both plants and mammals, and this protein is 99% identical to the mouse, rat and bovine orthologs. This gene contains a 7 bp repeat sequence in its 5' UTR, and changes in the number of this repeat has been associated with early-onset schizophrenia [26].

The 14-3-3 proteins are a family of conserved regulatory molecules expressed in all eukaryotic cells. A striking feature of the 14-3-3 proteins is their ability to bind a multitude of functionally diverse signaling proteins, including kinases, phosphatases, and transmembrane receptors. This plethora of interacting proteins allows 14-3-3 to play important roles in a wide range of vital regulatory processes, such as mitogenic signal transduction, apoptotic cell death, and cell cycle control [37]. Acronyms14-3-3 family proteins interact with many signaling molecules, such as MAPK kinase kinase, Raf-1, Wee1, Cdc25, cyclin B1, protein kinase C, IGF-I receptor, insulin receptor substrate 1, Bad, and Bcl [38-42], and regulate several signal transduction pathways [43-45]. Also, 14-3-3 proteins help two molecules to interact or to interrupt the association between two molecules by functioning as molecular scaffolds [46].

Binding of a protein to a 14-3-3 protein may result in stabilization of the active or inactive phosphorylated form of the protein, to a conformational alteration leading to activation or inhibition, to a different subcellular localization or to the interaction with other proteins. Currently genome- and proteome-wide studies are contributing to a wider knowledge of this important family of proteins [47].

The molecular consequences of 14-3-3 binding are diverse and only partly understood. Disturbance of the interactions with 14-3-3 proteins may lead to disease like cancer. In this study, gremlin 1 binds YWHAH protein in vitro and in vivo. We suspect that this binding may disturb the interactions with 14-3-3 proteins and lead to disease like cancer.

## Conclusion

Gremlin 1 was overexpressed in various human tumors and plays a oncogenic role especially in carcinomas of the cervix, lung, ovary, kidney, breast, colon, pancreas and sarcoma. Although further investigation with larger number of samples will be needed, these results indicate that increased expression of the PIG-2 may be associated with human tumorigenesis. Our study suggests that over-expressed gremlin 1 functions by interaction with YWHAH in human tumorigenesis. While further studies are needed to characterize cellular functions and regulatory mechanisms, gremlin 1 is a candidate oncoprotein in the development of many types of human cancers, and gremlin 1 and its binding protein YWHAH could be good targets for developing diagnostic and therapeutic strategies against human cancers.

## Competing interests

The author(s) declare that they have no competing interests.

## Authors' contributions

HN and JWK designed the project. SMS and HKK performed DDRT-PCR experiment. SH and GWC performed the expression profiling experiments. SYH and TEK performed yeast 2-hybrid, GST full down and immunoprecipitation experiments. All authors contributed to the writing of the manuscript and have read and approved its final draft.

## Pre-publication history

The pre-publication history for this paper can be accessed here:
